# Phylogenetic Properties of RNA Viruses

**DOI:** 10.1371/journal.pone.0044849

**Published:** 2012-09-20

**Authors:** Simone Pompei, Vittorio Loreto, Francesca Tria

**Affiliations:** 1 Complex Systems Lagrange Lab, Institute for Scientific Interchange (ISI), Torino, Italy; 2 Department of Physics, University of Torino, Torino, Italy; 3 Department of Physics, Sapienza University of Roma, Rome, Italy; University of Zaragoza, Spain

## Abstract

A new word, phylodynamics, was coined to emphasize the interconnection between phylogenetic properties, as observed for instance in a phylogenetic tree, and the epidemic dynamics of viruses, where selection, mediated by the host immune response, and transmission play a crucial role. The challenges faced when investigating the evolution of RNA viruses call for a virtuous loop of data collection, data analysis and modeling. This already resulted both in the collection of massive sequences databases and in the formulation of hypotheses on the main mechanisms driving qualitative differences observed in the (reconstructed) evolutionary patterns of different RNA viruses. Qualitatively, it has been observed that selection driven by the host immune response induces an uneven survival ability among co-existing strains. As a consequence, the imbalance level of the phylogenetic tree is manifestly more pronounced if compared to the case when the interaction with the host immune system does not play a central role in the evolutive dynamics. While many imbalance metrics have been introduced, reliable methods to discriminate in a quantitative way different level of imbalance are still lacking. In our work, we reconstruct and analyze the phylogenetic trees of six RNA viruses, with a special emphasis on the human Influenza A virus, due to its relevance for vaccine preparation as well as for the theoretical challenges it poses due to its peculiar evolutionary dynamics. We focus in particular on topological properties. We point out the limitation featured by standard imbalance metrics, and we introduce a new methodology with which we assign the correct imbalance level of the phylogenetic trees, in agreement with the phylodynamics of the viruses. Our thorough quantitative analysis allows for a deeper understanding of the evolutionary dynamics of the considered RNA viruses, which is crucial in order to provide a valuable framework for a quantitative assessment of theoretical predictions.

## Introduction

Investigating the relations between the evolutionary history of an organism, the forces that shape it and the resulting topological properties of its phylogenetic tree, is a major topic in evolutionary biology [1], [2], [3], [4], [5], [6], [7], [8]. Since the very first reconstructions of the *tree of life*, it was noticed the presence of an uneven distribution of sizes among different taxonomic groups [1]. The first attempt of explanation came from Yule [2], who proposed a very simple model of diversification in which, starting from a single species in the tree, every species can split into two new species with a uniform probability. The resulting genealogy of the Yule model (which is statistically equivalent to the so called ERM, Equal-Rate Markov Model [5]), displays asymptotic statistical properties which are the same of a *totally balanced tree* (i.e., a binary tree in which every node has exactly two branches). Since then, new models of diversification have been introduced [5], [7], [8], able to generate non-ERM phylogenies (i.e., with a higher level of imbalance) and different possible measures for assessing the degree of imbalance of a tree have been proposed [9], [4], [10], [11], [12], [13], [14], [15].

The properties of different indices of tree imbalance have been intensively studied in order to assess their ability in discriminating among the different speciation models [16], [17], [18], [6]. The effectiveness of the different indices in discriminating among different branching models has been often questioned. For instance Stich and Manrubia [14] observed that the evolutionary parameters only weakly affect the topological properties of phylogenies while the tree size seems to importantly affect the scaling exponents, leading to asymptotically balanced trees.

Qualitative differences on the shape of phylogenies of RNA viruses have been related to different selective pressure of the host immune system on the viral evolutionary dynamics [19]. The availability of massive sequences databases has triggered studies aimed at a deeper understanding of the so-called phylodynamics of RNA viruses [20], [21], namely the complex interplay between molecular evolution and the interactions among pathogens, the host immune system and the epidemic dynamics. RNA viruses, indeed, feature an exceptionally high nucleotide mutation rate, about one million times higher than that of vertebrates, which confers an outstanding adaptive ability on them. Their genes can accumulate observable genetic mutations on a time scale comparable to the responsiveness of the host immune system. Because of this high level of mutation rate, a viral population is not composed by identical clones, rather different viruses present closely related but not identical genotypes, differing in at least one nucleotide one from another. This high genetic heterogeneity, moreover, also results in the variability of phenotypes in the population, where the fitness is not evenly distributed among all the coexisting strains. This particular type of organization is called a *quasi-species*. Originally used by Manfred Eigen [22], [23] to model the evolution of the first macromolecules on earth, the quasi-species concept has been applied to populations of replicating molecules and RNA viruses [24], [25], [26], [27].

The relative fitness of the coexisting strains in the virus populations largely depends on the selection driven by the host immune system [19], through a mechanism referred to as cross-immunity. After being infected, the host can acquire immunity against different related strains of the same virus and this cause a differential survival ability among coexisting strains. Phylogenetic trees of RNA viruses store information about the relative fitness of each evolutionary lineage and the analysis of the tree shape provides an indirect investigation of the interaction between the host immune system and the virus.

**Figure 1:**
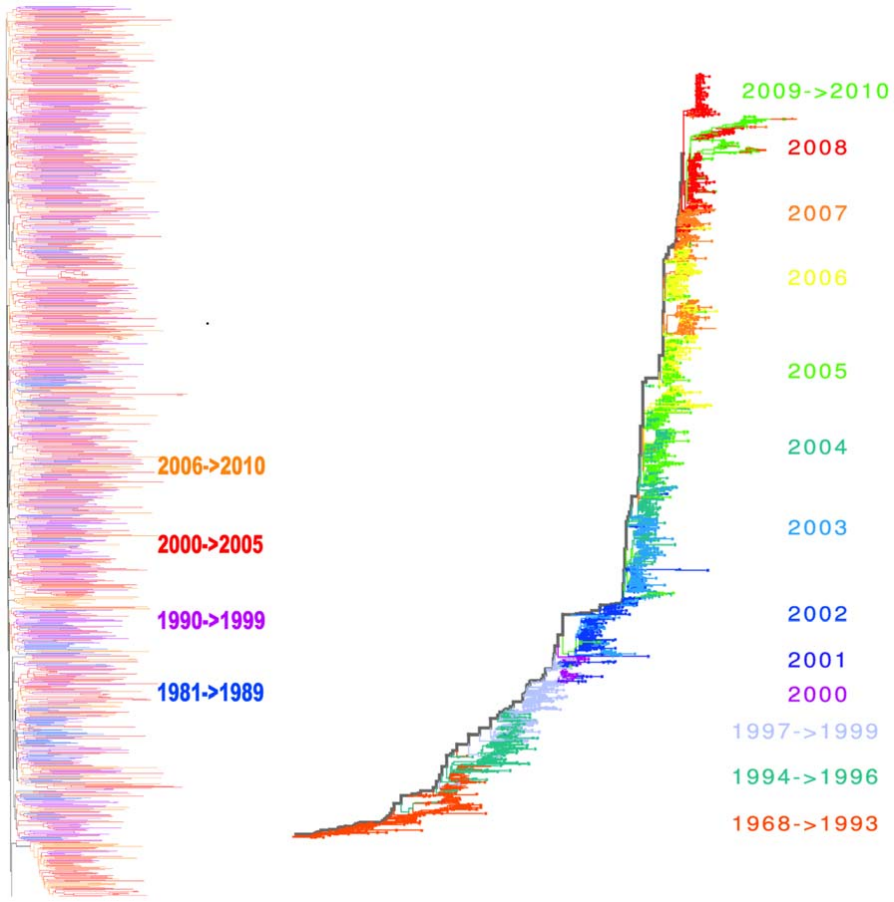
HIV Inter-Host and Human Flu H3N2 Phylogeny Trees. Inferred phylogeny of the HIV-1, subtype B virus at a geographical global scale (Env gene, region C2V5) (Left), and the Human Influenza A Virus (haemagglutinin gene HA), subtype H3N2, at the same scale (Right). Details on the inference method used are reported in the main text. In each phylogenetic tree, we use a color code to visually identify viruses isolated in the same temporal interval. For example, we colored in red all the branches of leaves isolated between 2000 and 2005 for the HIV-1 virus (left), while, for instance, blue is used in the phylogenetic tree of Human Flu (right) to mark all the leaves isolated in the year 2001. In the phylogenetic tree of the Human Flu, leaves isolated in the same temporal interval cluster in the same clade and there is just one evolutionary lineage, the so called *“Trunk”* (marked in grey), connecting all of them. These patterns are not observed in the phylogenetic tree of the HIV-1 virus. The tree of the Human-Flu H3N2, moreover, is visibly much more imbalanced with the respect to the HIV-1 one. Details on the definition of the imbalance level and its quantification are in the main text.

A particular interest has been raised for the evolutionary dynamics of the human Influenza A virus, due to the relevance of predicting new emerging strains for effective prevention strategies and timely vaccine preparation [28]. In this case, hosts acquire immunity against the strain of the primary infection, but can still be re-infected by different strains which have mutated at key antigenic sites. In this way, strains that have already circulated within the population can be prone to extinction, while the emerging new variants feature a higher survival ability, being able to re-infect hosts immune to earlier types. Because of the combination of the high level of transmission among hosts and hosts mobility, moreover, every year each viral epidemic is caused by one dominant strain circulating at a global scale. The shape of the inferred phylogenetic tree of the Human Influenza A virus [29], [30], [31], [28] reflects these properties. All the strains circulating in the same annual epidemic belongs to the same quasi-species, with similar antigenic properties, and cluster in the same group in the inferred phylogenetic tree. Different quasi-species follow one another in subsequent years, revealing the presence of a single path in the tree, usually called *trunk* (see Fig. 1). The trunk, moreover, has the highest rate of amino acid replacement at key antigenic sites, revealing a strong selective pressure [29]. Among the co-circulating strains, the one with the highest amino-acidic similarity with the trunk has the highest probability to become the dominant strain of the subsequent year, providing a basis to design annual vaccines [28]. From all said above, we expect that suitable imbalance measures performed on the Influenza A virus should reveal a high level of imbalance of the corresponding phylogenetic tree.

We provide here, for the first time to our knowledge, a quantitative assessment of the imbalance properties of the Influenza A phylogenetic tree, as inferred from sequences coding haemagglutinin (HA), a surface protein that constitutes the main target for the host humoral response. We compare these results with those obtained on the reconstructed phylogenetic trees of five other viruses, for whom a different selection pressure due to the host immune system has been hypothesized [19]. The aim of this study is twofold: on the one hand, we wish to provide the community with a set of observables, tested on real phylogenies, able to discriminate between different evolutionary processes; on the other hand, we aim at introducing, through these observables, a reference point through which different modeling schemes can be compared. In the last few years many models have indeed been introduced that mimic the viral evolution of the human Influenza A at the sequence level [32], [33], [34], [35]. Comparison with the natural viral evolution was performed so far in a qualitative way, while the availability of specific mathematical tools would increase the ability in discriminating between two or more competing hypotheses and, possibly, the predictive power of these models.

In this work we introduce a framework for the analysis of both topological and metrical properties of inferred trees of RNA viruses. We focus in particular on the topological properties, introducing a new methodology for the correct quantification of the degree of imbalance of the inferred phylogenetic trees. To this end, we consider the phylogenies of six RNA viruses, characterized by different selective pressure from the host immune system, namely the Human Flu H3N2 virus, the Avian Flu H5N1 virus, the Swine Flu H1N1, the Measles virus, the HIV-1 virus, both at the Intra-host and Inter-host level. The main features of their evolutionary dynamics will be described in the Methods section. We applied a *distance-based* method for the phylogenetic reconstruction, considering data-sets with thousands of sequences. For all the analysis, we used a recently introduced Stochastic Local Search algorithm, Fast-SBiX [36], [37].

The outline of the paper is as follows. We first give a detailed description of the features of the data-set we used for the inference of the phylogenetic trees, as well as of the inference strategy we adopted. We then recall the most relevant properties of the phylodynamics of the six viruses we took into consideration. Next we focus on the topological properties of the phylogenetic trees. We point out the limits of the quantification of the imbalance level attained by means of standard imbalance metrics [4], [16], [17], [10], [11], [12], [15], and then we introduce our new methodology. We show that our approach correctly quantifies the imbalance level of the six inferred phylogenetic trees we considered. With a slight variant of our methodology, moreover, we show a possible way to detect the *antigenic drift* [38] of the Human Flu H3N2 virus, through the analysis of the evolution of the imbalance metrics over time.

In File S1 we give a detailed description of all the standard imbalance metrics presented in [4], [16], [17], [10], [11], [12], [15]. Further, we present the probabilistic approach for the imbalance quantification, introduced in [14], and a variant we introduce in this context, pointing out the limits of this approach when applied to inferred phylogenetic trees of RNA viruses. Finally, we present an analysis of the metrical properties of the six trees, which we used to estimate the fixation rate of the genomic region of corresponding viruses.

## Materials and Methods

### Data-set and inference of the phylogenetic trees

Our data-sets include the genome sequences of the populations of three viruses of Influenza A (Human Flu H3N2, Avian Flu H5N1, Swine Flu H1N1), the HIV virus, both at intra-host and at inter-host level, and the Measles virus. Table 1 summarizes the main features of the data-sets used. For each virus we performed a multiple alignment of all the genome sequences considered, making use of the online version of MAFFT [39], which allows for fast and accurate alignment when dealing with large data-sets. Table 1 also reports the total lengths of the sequences after the alignment. In File S2 we report the accession numbers of the sequences in our data-sets. Pairwise distances have been computed according to the *Jukes-Cantor Model* [40]. All the phylogenies have been inferred making use of a recently introduced Stochastic Local Search algorithm *Fast-SBiX* [37], [36], whose accuracy in the phylogenetic reconstruction has been tested both on artificial data [37], [36], [41] and on language trees inference [42].

**Table 1:** Properties of the data-set used for the inference of the phylogenetic trees.

Virus	Genomic region (CDS)	N	Length	Source	reference sequence
Human Flu H3N2	Haemagglutinin (HA)	4227	1700	Influenza Virus Resource database [50]	Oldest sequence
Avian Flu H5N1	Haemagglutinin (HA)	1157	2167	Influenza Virus Resource database [50]	Oldest Sequence
Swine Flu H1N1	Haemagglutinin (HA)	315	1716	Influenza Virus Resource database [50]	Oldest Sequence
HIV-B Intra-Host	Env gene (C2V5)	282	846	HIV Sequence Database [43]	HIV-C sequence
HIV-B Inter-Host	Env gene (C2V5)	1023	1263	HIV Sequence Database [43]	HIV-C sequence
Measles Virus	N gene	985	581	GenBank [51]	Rinderpest Virus Sequence

In File S2 we report the accession numbers of the sequences in our data-sets.

The imbalance properties of a topology can only be defined for rooted phylogenies, while typically inferred trees are unrooted. We identified, for each data-set, a reference sequence as the common oldest ancestor, and we rooted the inferred phylogeny in the closest internal node of this leaf. When considering Flu viruses, because of the rapid turnover of the circulating strains over time, the oldest sequence of each data-set can be used as the reference sequence. In the analysis of the HIV virus, since we focused on the subtype B, we considered the same genomic region of a sequence of subtype C as a reference strain (as suggested in [43]). The N gene of a Rinderpest Virus, finally, was used as the oldest common ancestor for the Measles Virus data set [44].

### Phylodynamics properties of the six RNA viruses

The phylogenetic properties of the **Human Influenza A virus** H3N2 are deeply affected by its interaction with the host immune system. After recovering, hosts acquire immunity for the strain they have been infected, but they can still be re-infected by different strains which have mutated at key antigenic sites. In this way the circulating strains get continuously replaced by new variants that can re-infect hosts already immunized to earlier circulating strains. This turnover takes place on annual time scale and is responsible for the call for new vaccines to be formulated before each annual epidemic. The high level of transmission among hosts, combined with their elevated mobility, entails the emergence of a dominant strain circulating at a geographical global scale. All the dominant infective strains arisen in subsequent years, therefore, share the same evolutionary lineage.

The inferred phylogenetic tree of the haemagglutinin (HA) gene of human Influenza A virus, H3N2 subtype [29], [30], [31], [28], shown in Fig. 1, displays a strong temporal pattern. All the leaves isolated in the same year cluster in the same group. These temporal clusters are the fingerprint of the turnover of the coexisting strains. A single path in the tree, usually denoted as *trunk*, connects all the clusters. As already stressed, we expect the phylogenetic tree of the human Flu H3N2 to feature a high degree of imbalance, because of the uneven distribution of the survival ability among all the evolutionary lineages.

The populations of both the **Swine** H1N1 and **Avian** H5N1 **Influenza A viruses** are also under deep selection from the host immune system, displaying the same multi-strains dynamics of the human Influenza A virus. In this case, however, the reduced mobility of the hosts leads to the emergence of few independent evolutionary lineages, surviving in different geographic regions. Again, we expect the inferred phylogenetic trees of the haemagglutinin (HA) gene of these two viruses to be highly imbalanced, though featuring different co-existing lineages, corresponding to different geographic regions.

The **Measles Virus** features different immunological properties with respect to the Influenza A Virus. Along with a lower mutation rate with respect to Influenza (see File S1), in this virus all the mutated strains are not antigenically significantly different. This means that, after being infected, hosts acquire a lifelong immunity from all the other strains. The host immune system-driven selection is thus very weak. The introduction of the vaccine, in 1963, has significantly reduced the number of infections caused by this virus, even though seasonal outbreaks still occur in regions with low vaccination coverage. Due to the lack of a differential selection on the different lineages, phylogenetic trees of the measles virus, usually inferred from the sequences coding the N gene [19], is expected to display a very low degree of imbalance.

The dynamics of the **HIV virus** is characterized by persistent host infection. The **intra-host** evolution of the HIV Virus is very fast and takes place on a time scale of days. The continual and strong immune selection pressure, from either neutralizing antibodies or cytotoxic T lymphocytes, results in the extinction of many evolutionary lineages, with few ones prolonging the infection, which can last decades. The inferred phylogenetic tree for the HIV virus within a single host resembles the one of the Influenza A virus at a population level. We thus expect imbalance measures to detect a high degree of imbalance. The **inter-host** dynamics of the HIV virus, on the contrary, takes place on a scale of months or years, i.e., the typical time on which the transmission occurs. Multi strains coexist, being able to infect different individual hosts. The inferred phylogenetic tree of the *Env* gene (region C2V5), at the inter-host level, is thus expected to be very balanced. This phylogenetic tree (as shown in Fig. 1), moreover, do not feature any temporal pattern [19], since leaves do not cluster according to the year of isolation, while it can be used to trace the demographical and spatial history of transmission [45].

### Imbalance measures

**Figure 2:**
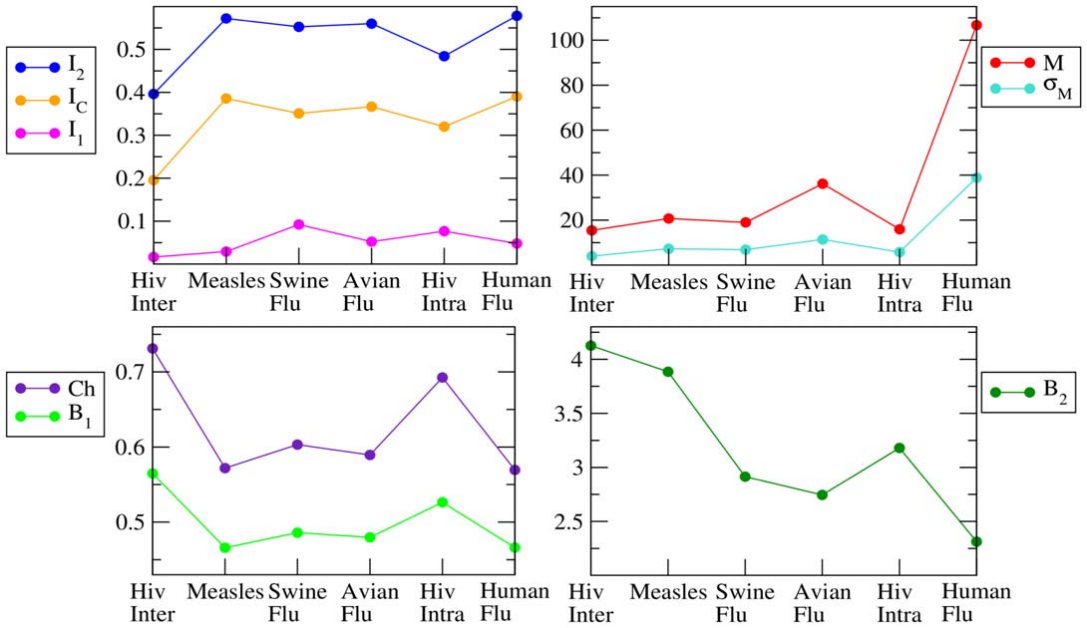
Quantification of tree shape imbalance with the metrics of **Table 2**. In this graph we show the values of the imbalance metrics defined in Table 2 for all the six inferred trees of RNA viruses: HIV Inter-Host, Measles Virus, Swine Flu H1N1, Avian Flu H5N1, HIV Intra-Host and Human Flu H3N2. Phylogenies in the x-axis have been ordered according to increasing values of the expected degree of imbalance (see main text for details).

Different indices have been introduced in the literature [4], [16], [17], [10], [11], [12], [15] in order to quantify the asymmetry of a topology i.e., the uneven distribution of the number of leaves between left and right branches, and their ability in differentiating simple models of diversification has been analyzed [17], [16], [18], [7], [8]. In Table 2 we report the definition of the most commonly used imbalance metrics, together with their values on a totally balanced and on a totally imbalanced tree, referring to the File S1 for a thorough discussion. In fig. 2 we show the values each imbalance metrics takes on the phylogenetic trees of the six considered RNA viruses. We see that different metrics often give conflicting information, such that a ranking for the imbalance level of the six phylogenetic trees does not clearly emerge from this analysis. In fact, extracting information on the imbalance level of a tree from the bare number as given by the above defined metrics can be misleading due to the fact that, with the exception of 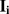 (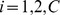), all the metrics explicitly depend on the size 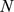 of the considered tree.

**Table 2:** Imbalance Metrics.

Metrics	Formula	Totally Balanced	Totally Imbalanced
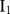	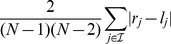	0	1
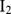	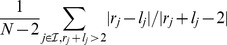	0	1
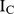	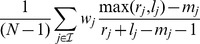	0	1
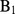	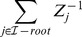	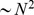	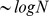
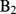	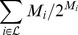	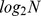	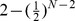
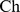	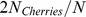	1	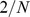
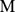	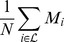	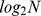	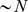
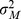	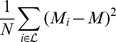	0	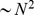

In this table we report the definition of eight imbalance metrics defined in the literature [4], [16], [17], [10], [11], [12], [15]. The last two columns show the values of such metrics for totally balanced or totally imbalanced trees with 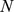 leaves. 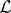 is the set of all the leaves in the tree while 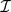 is the set of all the internal nodes. For each internal node 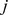, 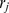 and 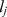 are respectively the number of leaves belonging to the right and left subtrees descending from 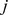, and we define 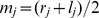 and 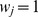 if 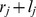 is even, 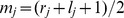 and 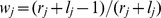 if 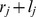 is odd, 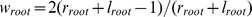. 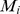 is the topological distance between leaf 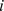 and the root i.e., the number of internal nodes in the path connecting 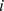 to the root. Given an internal node 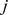, 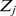 is the maximum topological distance between 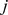 and one of its descending leaves. 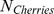 is the number of cherry leaves within the tree, where a cherry is a couple of leaves which descend from the same internal node. We refer to the File S1 for further details about the definition of the imbalance metrics.

### A new comparison strategy

In order to compare trees of different sizes, and to deal with finite-size effects, a careful analysis is in order, where the dependence on the system size (number of leaves) 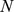 is taken into account. We here introduce a new methodology and we use it to quantify the imbalance level of the inferred phylogenetic trees of the six RNA viruses. We shall specifically consider the following three metrics:

***M*** **.** The *mean topological distance* [12] 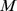 of the leaves from the root, defined as:
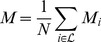
(1)where the sum runs over the 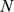 leaves of the tree and 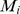 is the topological distance of the 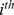 leaf from the root i.e., the number of nodes in the path connecting them. When considering a totally balanced tree, 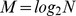 since all the 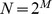 leaves share the same topological distance from the root. On the other hand, for a totally imbalanced tree, 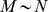, i.e., 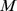 exhibits an asymptotic linear behavior, since in this case 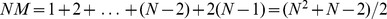.

***D*** **.** The *mean depth* 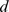 [13], i.e., the mean topological distance of each node (internal nodes and leaves) from the root, defined as:
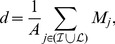
(2)where the sum runs over all the nodes of the tree (leaves and internal nodes) and 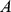 is the tree size defined as the total number of internal nodes and leaves of the tree: 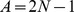 in a rooted binary tree. Here 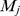 is the topological distance of the 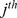 element of the tree (leaf or internal node) from the root. In a totally balanced tree, the leading contribution for the mean depth 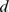 at large sizes is logarithmic: 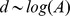, while in a totally imbalanced one 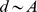 i.e., it displays a linear asymptotic behavior [13]. We remark that the metrics 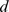 is considered as a function of 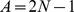. During the following discussion, for the sake of simplicity, we will address a generic metrics 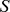 as a function of 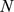, implying that, whenever 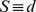, then 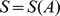.

***I'*** **.** The *Asymmetry* metrics is inspired by the definition of 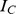 but we consider it unnormalized, as:
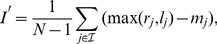
(3)where the sum runs over each internal node 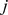 of a rooted tree, 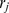 is the number of leaves descending from the right branch of 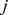 and 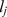 the number of leaves descending from its left branch, and 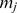 is the smallest integer not smaller than 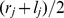. The definition in (3) is such that 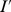 is constantly null on a totally balanced tree and 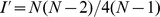 on a totally imbalanced one.

The methodology we use here consists in exploring sub-trees of all the possible sizes within a topology. In particular, let 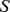 be any of the three metrics defined above (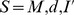), and let 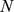 be the total number of leaves in the phylogeny under investigation. Our methodology consists in the following steps:

1. We randomly select 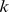 independent sets of 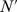 (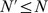) leaves in the phylogenetic tree, and extract, from the complete tree, the relative binary sub-trees induced by each set. (We remark that this is in principle different than reconstructing a new tree from the subset of 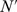 leaves. We checked however that the two procedures give the same quantitative results. We thus safely used the procedure described here, for the sake of computational economy).
2. For each of the 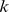 sub-trees extracted, we compute the imbalance level 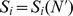, (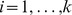), by means of the metrics 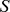, and consider the average 
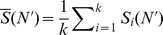
. The value of 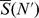 is thus an average value of the imbalance level of the phylogenetic tree at the size 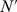.
3. We repeat the steps 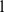 and 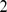 for each possible size 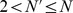.

The resulting curve 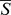 is thus a quantification of the average values for the index 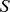, for all the possible sizes 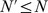. With our sampling procedure of subtrees within a tree, we can thus investigate the dependence of the imbalance metrics on the size of considered subtrees, and, at the same time, the sampling of many subtrees with the same size allows for a better statistical analysis of their imbalance level, in order to reduce the effect of noise and fluctuations.

A quantitive comparison of the imbalance properties of different phylogenies can thus be attained in two different and complementary ways. On the one hand, the relative position of the curves 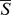 of different phylogenetic trees quantifies their relative imbalance level at the same size. On the other hand, we can explicitly consider the asymptotic behavior of the curves 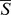, in order to be able to make a comparison with hypothesized evolutionary models.

The asymptotic behavior of 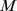, 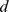 and 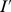 is analytically known in the cases of totally balanced or imbalanced trees. Further, the so-called beta-splitting model [5], [6] (a non-constructive model for phylogenies generation) characterizes the asymptotic behavior of the above balance metrics also in intermediate cases. An asymptotic behavior as 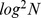 is found to correspond to a well defined value of the 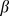 parameter in the beta-splitting model and it is associated to a great variety of real phylogenetic tree [5]. Moreover, in some recent analysis [13], [8], authors found the same asymptotic behavior (respectively 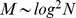 and 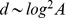) for the mean topological distance 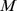 and the mean depth 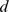 when looking at the scaling laws of all the trees in the PANDIT [46] and TreeBASE [47] databases. It should be noted that logarithmic exponent can greatly vary in these cases and the authors of [8] mention that a power law 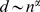 with 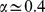 fits TreeBASE data equally well [48] while for larger tree sizes as those contained in PANDIT, the 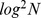 seems more accurate.

Taking these results as important hints we here adopt our resampling strategy described above to fit the asymptotic behavior of the imbalance metrics 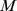, 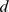 and 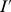 with the function:
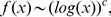
(4)where 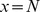 when considering 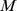 and 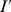, and 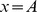 when considering 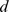. The exponent 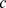 would be 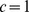 for a fully balanced tree and 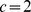 in the beta-splitting model with the parameter 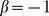 (the AB model [5]).

## Results

**Figure 3:**
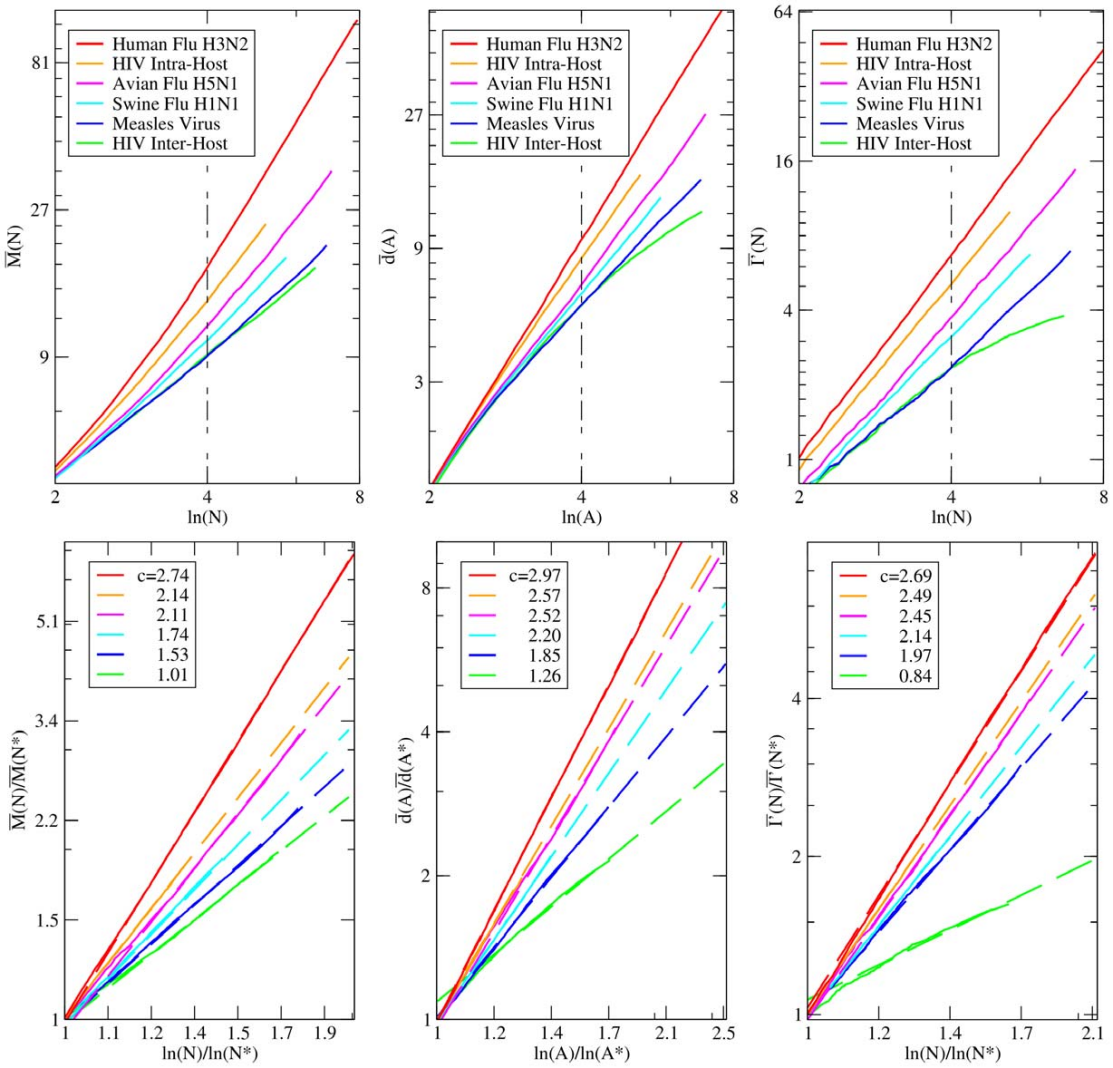
New comparison strategy results. Top line: Mean topological distance from root to leaves (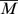), mean depth (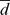) and asymmetry metric 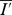 for all the inferred phylogenetic trees we considered, computed with the methodology discussed in the main text. Vertical dashed lines mark sizes 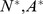. In the bottom lines we report the rescaled values for all the curves, where we compare the asymptotic imbalance level of the phylogenies isolating the linear trend of the curves for 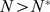 and 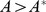, revealing in this way the asymptotic behavior with the functional form 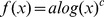. Solid lines are rescaled values of 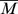, 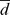 and 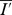 while dashed lines are for the relative extrapolations. In the inset we report the numerical values of 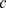 for each phylogeny.

In Fig. 3, top line, we report the results of the analysis of the imbalance level of the six phylogenies considered (Human Flu H3N2, Avian Flu H5N1, Swine Flu H1N1, HIV virus, both at Intra-host and at Inter-host level, and the Measles virus), obtained through our comparison strategy. For any metric 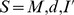, we computed the curve 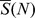, i.e. the average level of imbalance for all the possible subset sizes within the phylogeny. Notice that now we refer to N as the subset size of leaves considered. Data are shown as a function of 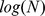 (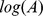 for 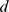) and in a double logarithmic scale graph; with this choice any function in the form 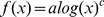 will be displayed as a straight line, with slope 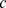.

The first quantification of the relative degree of imbalance of the phylogenies is given by the relative position of the corresponding curves 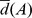, 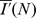 and 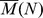, i.e. through a comparison of the relative imbalance level at the same size. From this perspective, a clear rank emerges, reflecting the expected hierarchy according to previous qualitative analysis [19]. In particular, from the most imbalanced tree to the most balanced one, all the three metrics reported in Fig. 3 recover the following order: Human Flu H3N2, HIV Intra-Host, Avian Flu H5N1, Swine Flu H1N1, Measles virus, HIV Inter-Host.

A further quantification of the absolute and relative degree of imbalance can be attained considering an extrapolation for the asymptotic behavior of these curves. All of them display a quite evident linear trend, when considering sizes 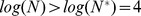 and 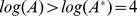, revealing an asymptotic trend with the functional form 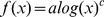. Results are shown in bottom line of Fig. 3, where we also report the numeric values of the 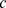 exponent for each phylogeny, which allow for a quantitative comparison of their asymptotic balance properties. Again, the arising hierarchy is shared by all the three metrics considered and, moreover, is exactly the same as the one deduced by the relative position of the curves. In addition, when considering 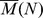 and 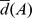, the value 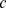 is an absolute assessment of the deviation from a totally balanced tree, for which 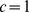. From this point of view, the HIV Inter-Host phylogeny turns out to be an almost totally balanced tree (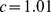 for 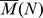 and 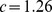 for 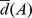). On the other hand, the limit of the totally imbalanced tree (for which 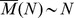 and 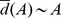) is never approached by all the phylogenies we considered. This can be intuitively explained considering that the selective pressure, when is present, although fast enough to avoid proliferation of antigenically different strains, acts on a longer time scale with respect to the time scale of mutations. As already stressed in the introduction, the viral population in a single instant is represented by a quasi-species rather than a single strain. This reflects on the phylogenetic tree, hiding its the global imbalance. In other words, at a small (yearly) time scale, the phylogenetic tree looks balanced, being formed by closely related strains belonging to the same quasi-species, while on a higher time scale (many years) selection takes place and the presence of a well defined trunk and of an uneven survival distribution is clearly visible. In order to fully appreciate these two different time scales, in the next section we shall present a further analysis of the evolution of the imbalance metrics over time.

It is worth to remark that all the three imbalance metrics we have considered consistently point out to the same hierarchy of the degree of imbalance of the six phylogenies analyzed. According to this classification, the phylogenies of the FLU viruses and the phylogeny of the HIV Intra-host virus turn out to be the most imbalanced, while the HIV inter-Host virus and the Measles virus phylogenies appear to be the most balanced ones. These findings suggest that, in order to discriminate between different level of imbalance due to different selective pressure, it is crucial to carefully quantify the imbalance level of sub-trees within the reconstructed phylogenies, considering then the balance properties as a function of the tree size.

### Imbalance properties as a function of time

The main feature of our approach is that of extracting a subset of leaves in a random way and study the properties of the relative sub-tree. The computation of the average properties of such sub-trees, as we have just shown, turned out to be useful to compare different phylogenies. It is important to remark that so far we did not fully exploit the information carried out by the phylogenetic tree. For instance we did not exploit the information about the years of isolation of the different strains.

One of the marking property of the phylogeny of the Influenza A virus, besides that of being imbalanced, is indeed that of displaying a strong temporal pattern, where the leaves cluster according to their year of isolation, and also to display a *‘trunk’* (see Fig. 1). This peculiarity is the signature of the *antigenic drift* [38], which is responsible for the call for new vaccine to be formulated before each annual epidemic. In order to perform a deeper investigation of the properties of such clusters we therefore introduce here a slight variant of our methodology.

**Figure 4:**
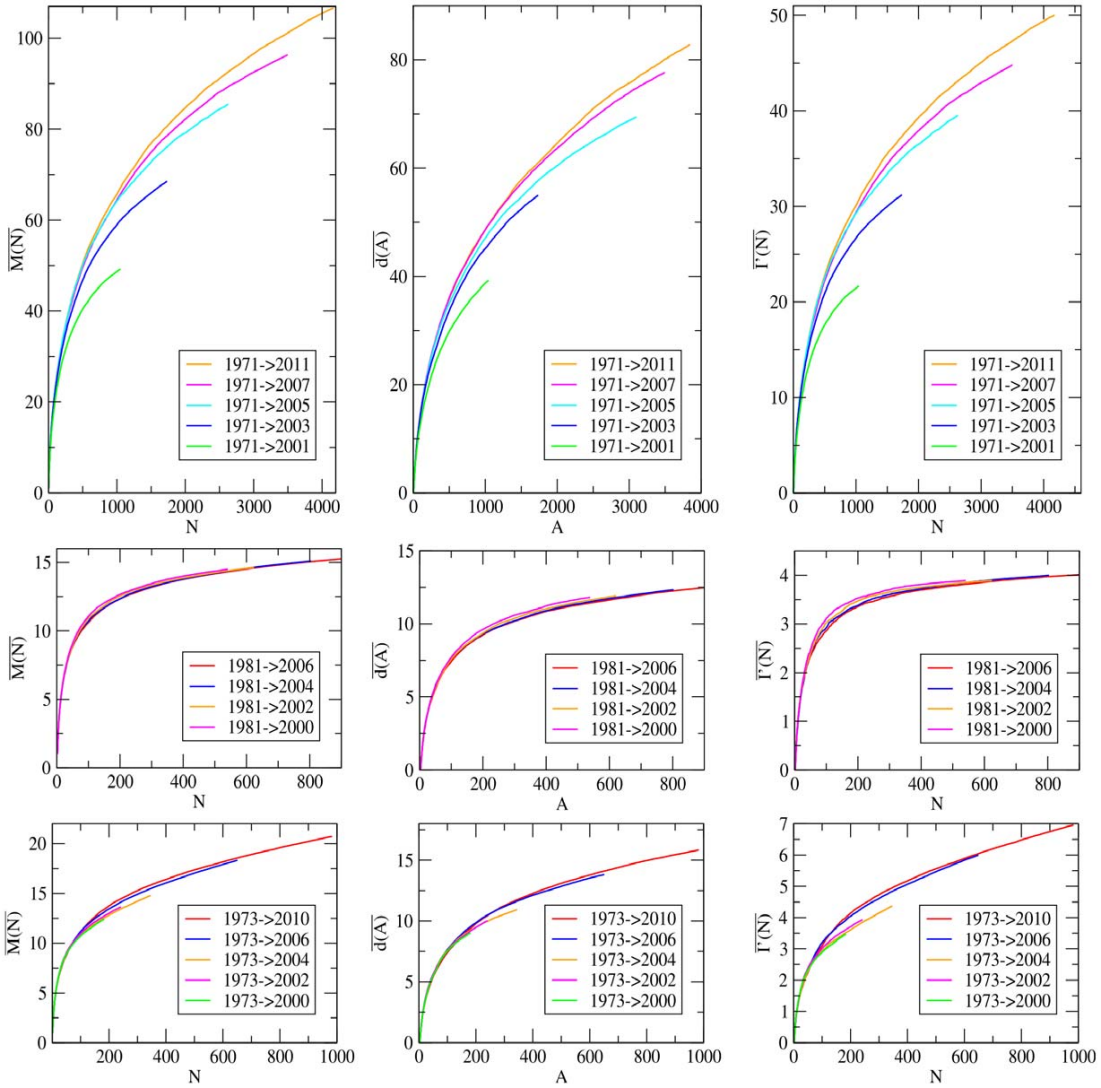
Relative behavior of 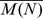, 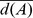 and 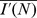 for subtrees of different time intervals. We focus here on the phylogenies of three RNA virus phylogenetic trees (Human-Flu H3N2 (top), HIV Inter-Host (middle), Measles virus (bottom),) considering the imbalance properties of their sub-trees, extracted with a temporal criterion. Each sub-tree is associated to a temporal interval (reported in the legend of the pictures), reflecting the years of isolation of its leaves. For example, in the case of Human Flu H3N2, the curve 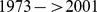 refers to the sub-tree enclosing all the strains, in our data-set, isolated between 1973 and 2001. For each sub-tree in this set we show the mean topological distance (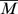), the mean depth (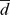) and the asymmetry 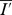 computed with the methodology presented in the main text. Asymptotic extrapolations of these curves are reported in the File S1.

We now consider a partition of the phylogeny investigated into a set of sub-trees, chosen with a temporal criterion. Each sub-tree is associated with a temporal interval and encloses all the leaves in the phylogenetic tree which have been isolated in such an interval (see also Fig. 4). For each sub-tree in this set, we measure the mean topological distance 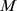, the mean depth 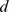 and the imbalance metrics 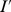, with the methodology described above. In Fig. 4 we report the result of this analysis for three phylogenies: Human-Flu H3N2, which features a strong temporal pattern, the HIV Inter-Host and Measles viruses, as a reference point of phylogenies without a marked temporal pattern [19].

In the Measles and HIV Inter-Host phylogenies the average values of the three imbalance metrics are about the same for each sub-tree. This suggests that the imbalance level of these two phylogenies do not depend on the year of isolation of the leaves considered or, equivalently, that sampling leaves with a temporal criterion is equivalent to a random sampling. This is a further evidence of the absence of temporal patterns within both Measles and HIV Inter-Host phylogenies.

In the Influenza A virus phylogeny, the imbalance properties of the sub-trees clearly depend on the corresponding temporal interval of the year of isolation of their leaves. In particular, the degree of imbalance increases when considering sub-trees corresponding to longer time intervals. Again, this is a further evidence for the existence of two different time scales characterizing the dynamics of this virus. At a yearly time scales, where all the strains are likely to belong to the same quasi-species, the diversification process is not deeply affected by the host-driven selection. This reflects in the low imbalance level of the corresponding sub-trees. On time scales of many years the driving force of the evolution is the selection induced by the host immune system and many quasi-species follow one another; this particular dynamics is detected by the increasing trend of the imbalance level.

The asymptotic behavior of the mean topological distance 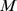, the mean depth 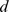 and the asymmetry index 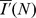 of the temporal sub-trees, moreover, attest the increasing trend for the imbalance level of the Influenza A virus phylogeny, and a very mild dependence for both Measles and HIV Inter-Host phylogenies (see File S1 for further details).

## Discussion

In the present paper we provided a framework for the quantification of the imbalance level of phylogenetic trees inferred from data sequences. Previous analysis of standard balance/imbalance metrics [14], [6] highlighted the difficulty in discriminating the imbalance level of phylogenies both inferred from real data and generated by evolutionary models. However, qualitative differences in the shape of reconstructed phylogenetic trees of different viruses have been highlighted, as a signature of different epidemiological features [19].

The methodology presented here makes use of a sampling procedure that allows to perform statistical analysis on a single phylogenetic tree. In this way, the dependence of the value of the imbalance metrics on the number of considered taxa can be investigated, and this turns out to be the relevant factor in order to quantify imbalance. The low computational cost of our approach, moreover, allows for a large statistical analysis of the imbalance level at a given size, through the sampling of many subtrees with the same number of taxa, in order to reduce the effect of noise and fluctuations. As all the statistical approaches, on the other hand, the significance of the analyses can be deeply affected by the size of the system, so the inference of phylogenetic trees with large number of leaves (thus data-sets of hundreds of sequences) is in order.

We presented here a detailed analysis of the topological properties of the phylogenetic trees of six RNA viruses inferred from nucleotide data sequences. In particular, we used the part of the genome that codifies for proteins more directly involved in the host immune response, in order to highlight the effect of selection on the evolutionary dynamics.

Remarkably, we were able to recover and quantify the relative degree of imbalance of each phylogeny, as both previously pointed out through qualitative observations and as expected from reasoning related to the selective pressure mediated by the host immune system. This is an important result since the the quasi-species nature of the viral population is in general a confounding factor for a proper quantification of the phylogentic tree imbalance. Taking into account this peculiary of the viral populations, we further provided a framework to discriminate phylogenetic trees whose shape is characterized by the presence of a trunk, like the Human Flu H3N2, from phylogenies in which this pattern is absent (Measles Virus, HIV Inter-Host).

Due to the proliferation of models aimed at explaining the peculiar evolutionary dynamics of Influenza A virus, we believe that a quantitative characterization of the imbalance properties of such a virus is crucial in order to discriminate among predictions corresponding to different evolutionary processes. The aim of this work is that of contributing to such a quantitative analysis, providing both a general mathematical and algorithmic framework as well as a demonstration of its implementation in the concrete case of discriminating among different RNA viruses featuring different levels of imbalance. Further works devoted to a thorough investigation of the predictions of the proposed methodology on artificial models where imbalance is due to different selective pressures, for instance neutral selection due to population bottlenecks [49], could shed light on how different driving forces behind evolution reflect on the phylogenetic tree topology.

## Supporting Information

File S1 **Detailed description of all the standard imbalance metrics** **[4]**, [16], [17], [10], [11], [12], [15], **[14]** **.** Analysis of the metrical properties of the six phylogenetic trees, which we used to estimate the fixation rate of the genomic region under analysis of each virus. Supplementary information on the extrapolation of the curves presented in Fig. 4.
(PDF)

File S2 **Accession number of the sequences in our data-sets.**
(TXT)
