# Supplementary material for: Phylogenetic Properties of RNA Viruses
Source: PLoS One. 2012 Sep 20;7(9):e44849. doi: 10.1371/journal.pone.0044849 (PMC3447819; doi:10.1371/journal.pone.0044849)
Supplement: File S1 — Detailed description of all the standard imbalance metrics [4] , [16], [17], [10], [11], [12], [15], [14] . Analysis of the metrical properties of the six phylogenetic trees, which we used to estimate the fixation rate of the genomic region under analysis of each virus. Supplementary information on the extrapolation of the curves presented in Fig. 4. (PDF) [file pone.0044849.s001.pdf]

Supporting Information for

Phylogenetic properties of RNA viruses

by Pompei et al.

Imbalance Metrics

In this paragraph we explain the main criteria used for the definition of the Imbalance Metrics we considered in our analysis [1–7].

The easiest way to estimate the degree of imbalance of a phylogenetic tree is to consider the asymmetry of internal nodes. Given an internal node j, one considers the difference between the number of leaves descending from the right/left branch of such node (r_j, l_j); both the asymmetry metrics $\mathbf{I_1}$ and $\mathbf{I_2}$ [4, 5] are based on this criterion. One then considers a weighed average of this difference over all the internal nodes, where the normalization term is such that the final score for $\mathbf{I_1}$ and $\mathbf{I_2}$ is equal to one for a totally imbalanced tree and is null for a totally balanced tree. The two metrics $\mathbf{I_1}$ and $\mathbf{I_2}$ give different relative weights to each internal node:

$$\begin{aligned}\mathbf{I_1} &= \frac{2}{(N-1)(N-2)} \sum_{j\in\mathcal{I}} |r_j - l_j| \\ \mathbf{I_2} &= \frac{1}{N-2} \sum_{j\in\mathcal{I}, r_j+l_j>2} |r_j - l_j| \,/\, |r_j + l_j - 2|.\end{aligned}$$

In $\mathbf{I_1}$ the normalization term is the same for all the internal nodes and is equal to the maximum value of the sum of all the differences $|r_j - l_j|$ in a tree with N leaves, which occurs in a totally imbalanced tree, its value being $(N-1)(N-2)/2$. The contribution of an imbalanced node j_{imbal} depends on its position on the tree. The more j_{imbal} is close to the root, the larger is the term $|r_{j_{imbal}} - l_{j_{imbal}}|$, since nodes close to the root have larger descending subtrees. This bias is removed in $\mathbf{I_2}$, where to each internal node is given a normalization term which properly reflects its position in the tree. In $\mathbf{I_c}$ [4, 5] a different quantification of the degree of imbalance of each internal node is introduced:

$$\frac{1}{(N-1)} \sum_{j\in\mathcal{I}} w_j \frac{\max(r_j, l_j) - m_j}{r_j + l_j - m_j - 1}. \tag{1}$$

Here one focuses on the difference between $m_j = (l_j + r_j)/2$, and the maximum value between r_j and l_j. The average is then computed considering a node-dependent weight, in order to remove biases like those present in $\mathbf{I_1}$ (with the correction term w_j for odd/even values: $w_j = 1$ if $r_j + l_j$ is even and $w_j = (r_j + l_j - 1)/(r_j + l_j)$ if $r_j + l_j$ is odd), the result being a metrics that, again, assigns a score equal to one to totally imbalanced trees and zero to totally balanced ones.

Another approach for the quantification of imbalance is based on the topological distance between pairs of internal nodes (j_1, j_2), i.e. the number of internal nodes in the path connecting j_1 and j_2. The indirect effect of increasing the degree of imbalance of an internal node j in a binary tree, indeed, is that of increasing its topological distance from all its descending nodes. Two metrics consider the topological distance, M_i, of a leaf i to the root:

$$\begin{aligned}\mathbf{M} &= \frac{1}{N} \sum_{i\in\mathcal{L}} M_i \\ \sigma^{\mathbf{2}}_{\mathbf{M}} &= \frac{1}{N} \sum_{i\in\mathcal{L}} (M_i - M)^2\end{aligned}$$

The mean topological distance $\mathbf{M}$ [6] and the standard deviation $\sigma^{\mathbf{2}}_{\mathbf{M}}$ [6] are the evaluations of the probability distribution of the M_i values in the tree. Two more imbalance metrics, $\mathbf{B_1}$ and $\mathbf{B_2}$ have been introduced in [7], within this approach:

$$\begin{aligned}\mathbf{B_1} &= \sum_{j\in\mathcal{I}-root} Z_j^{-1}\\ \mathbf{B_2} &= \sum_{i\in\mathcal{L}} M_i/2^{M_i}.\end{aligned}$$

In the imbalance metrics $\mathbf{B_1}$, for each internal node j, one considers the leaf with the larger topological distance from j: we call this distance Z_j. $\mathbf{B_1}$ is than the sum of all the inverse values Z_j^{-1}. For a given position in the tree j_{fixed}, the higher the degree of imbalance of the node j_{fixed} the larger the value of $Z_{j_{fixed}}$. The contribution of each node to $\mathbf{B_1}$, like in $\mathbf{I_1}$, depends on its position in the tree, since Z_j will be larger for nodes close to the root. The index $\mathbf{B_2}$ [7], is a weighted average of M_i, where each leaf is given a weight which reflects its position in the tree, the closer the leaf to the root, the higher its weight.

The fraction of cherry leaves **Ch**, finally, is another indirect measure of the degree of imbalance for binary trees:

$$\mathbf{Ch} = 2N_{Cherries}/N.$$

A couple of leaves define a cherry if both descend from the same internal node. In totally balanced trees with N leaves, all the leaves are grouped in $N/2$ cherries, while a totally imbalanced tree has only one cherry.

The subtree size (A) and the cumulative branch size (C)

In this paragraph we take into account two measures of imbalance recently presented in [8, 9] for a probabilistic definition of the imbalance level of a phylogeny. In a totally balanced tree, each node has exactly two subtrees with the same size and the number of subtrees of s is exactly half the number of those of size $s/2$. In a totally imbalanced tree, on the contrary, there is just one subtree for each size $s > 1$ (i.e., subtrees with more than one leaf), and there are $N - 2$ with only one leaf. The distribution of the sizes of the subtrees within a topology is then another possible marker for its level of imbalance.

We consider here two different measures for the size of each subtree, the subtree size A_i i.e., the total number of nodes (leaves or internal nodes) of the subtree rooted in i, and the cumulative branch size $C_i = \sum_{j\in\mathcal{I}_i} A_j$, where $\mathcal{I}_i$ is the set of all the internal nodes of such subtree. The probability distributions of all the values A and C in a phylogeny may display power low tails, $P(A) \sim A^{-\alpha}$ and $P(C) \sim C^{-\gamma}$. In addition, if a relationship exists between A and C of the form $C \sim A^{\eta}$, it holds $\eta = (1-\alpha)/(1-\gamma)$. The values of these tree exponents α, γ, η characterize the degree of the asymptotic imbalance of a tree. For instance for totally balanced tree $\alpha = 2, \gamma = 2, \eta = 1$, for totally imbalanced ones $\alpha = 0, \gamma = 1/2, \eta = 2$, while *Critical Branching Trees* [10] display $\alpha = 3/2$.

Phylogeny	α	γ	η
**Totally balanced tree**	2	2	1
**Totally imbalanced tree**	0	0.5	2
HIV Inter-Host	1.513	1.302	1.525
Measles Virus	1.511	1.304	1.529
Avian Flu H5N1	1.511	1.303	1.526
Human Flu H3N2 ($A < 400$,$C < 10000$)	1.487	1.293	1.628
Human Flu H3N2 ($A > 400$,$C > 10000$)	~ 0	0.624	1.824

Table S1. α, γ, η numerical values for $P(A)$, $P(C)$ and $C(A)$ curves of Fig. S1.

The probability distribution $P(A)$, $P(C)$ and $C(A)$ for four RNA virus phylogenies (Human Flu H3N2, Avian Flu H5N1, Measles Virus and HIV Inter-Host), are shown in Fig. S1. Two of these phylogenies have high expected level of imbalance (Human Flu H3N2 and Avian Flu H5N1), whereas for the other two phylogenies (Measles Virus and HIV Inter-Host) we expect a low level of asymmetry in the topology. In order to perform a fair comparison, we considered inferred trees with the same size ($N = 800$). To have a more robust statistical analysis, moreover, we examine 100 independently inferred trees, with a subset of 800 sequences for each virus.

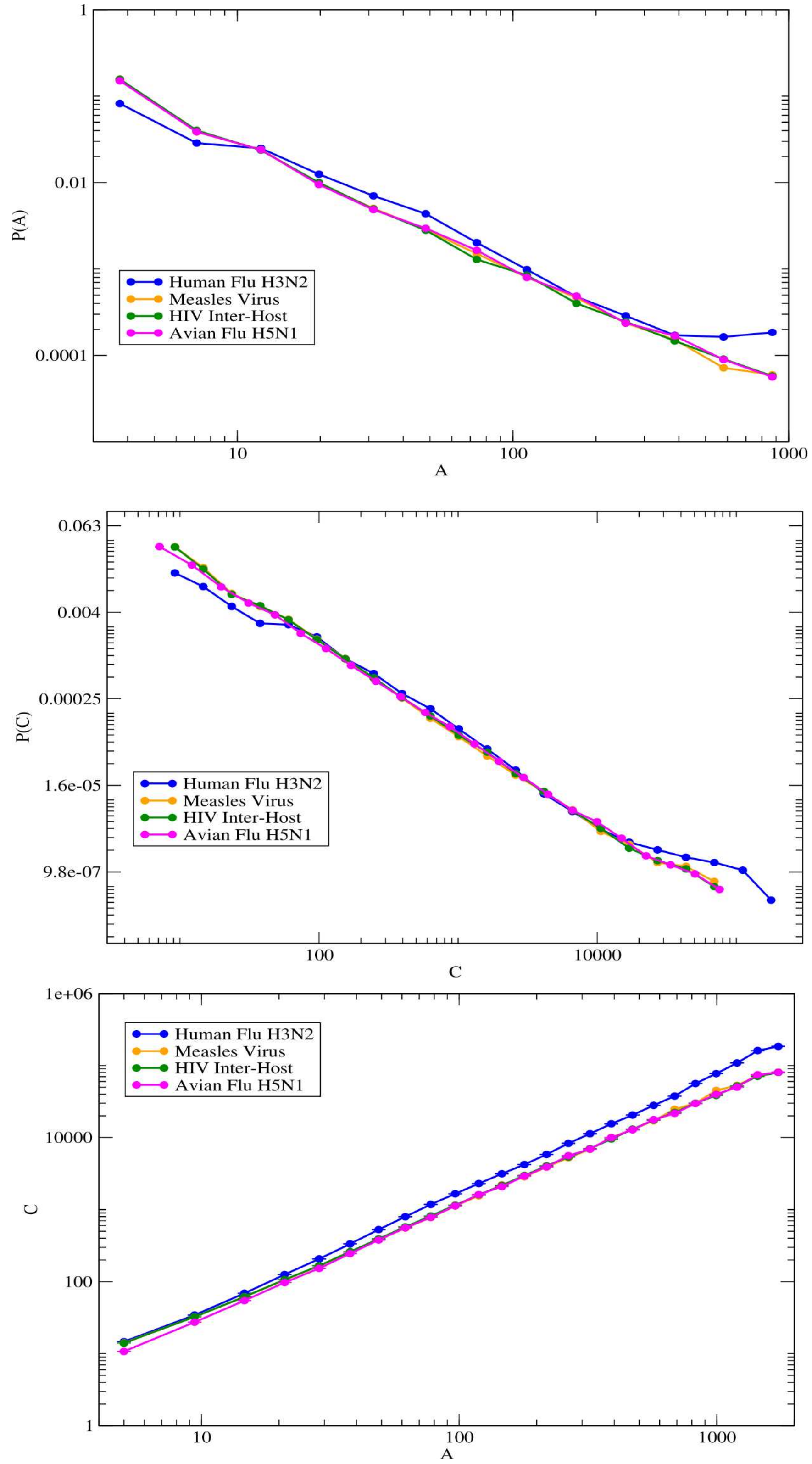

Figure S1. Subtree size (A) and the cumulative branch size (C) Probability distribution of subtree size ($P(A)$) and cumulative branch size ($P(C)$), and $C(A)$, for the phylogenies of four RNA virus tree: HIV Inter-Host, Measles virus, Avian Flu H5N1, Human Flu H3N2. For each phylogeny, we inferred 100 trees with $N = 800$ leaves, making use of 800 randomly extracted sequences of our data-set. In the case of Human Influenza A phylogeny $P(A)$, $P(C)$ and $C(A)$ exhibit a change of their curvature for $A > 400$ and $C > 10000$.

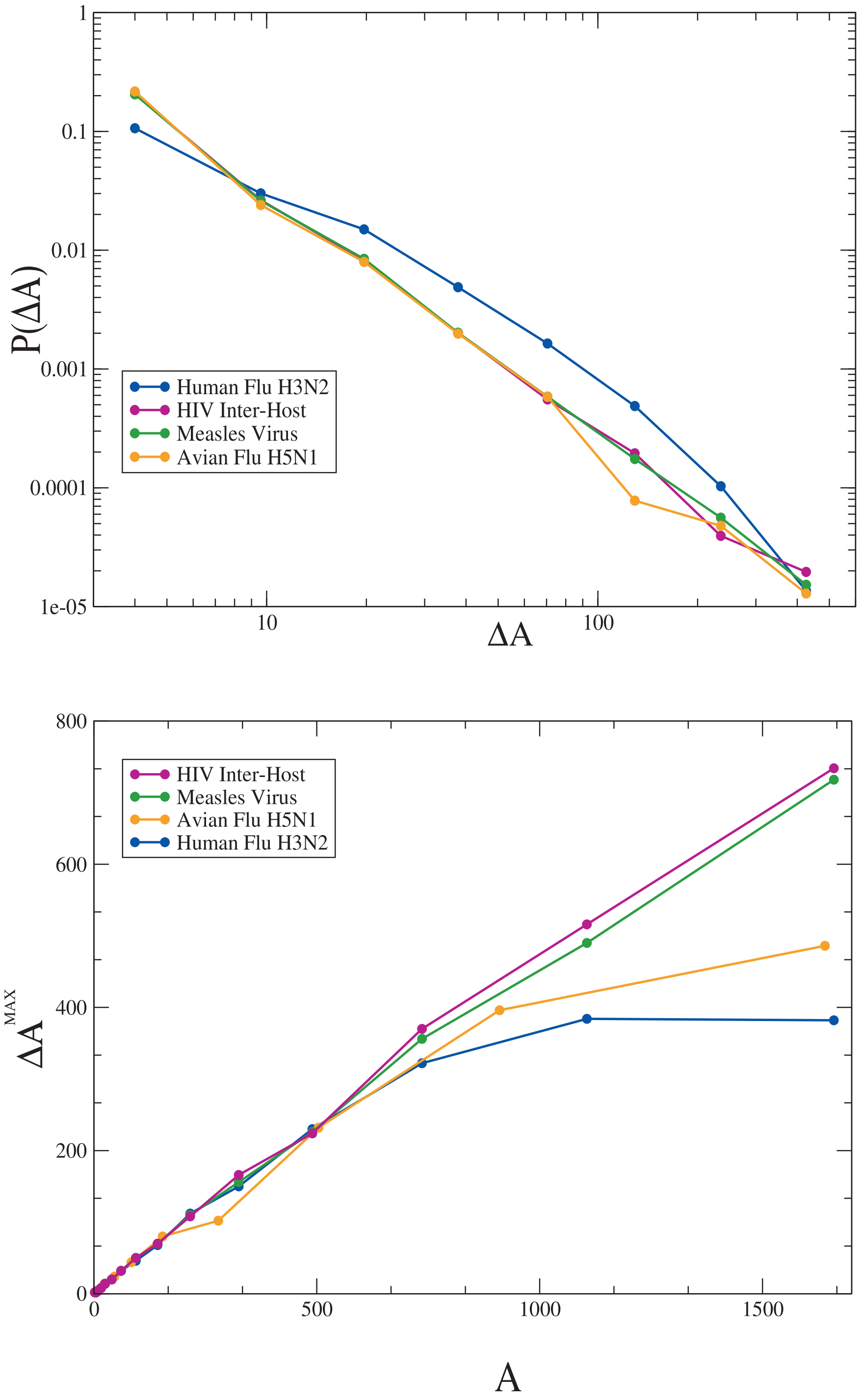

Figure S2. Variation of the Subtree Size (A) and the Cumulative Branch Size (C) In this graph we show the probability distribution of the Variations of the Subtree Size ($P(\Delta A)$)(top) and the correlation between ΔA^{Max} and A (bottom)(see text for definitions), for the phylogenies of four RNA virus tree: HIV Inter-Host, Measles, Avian Flu H5N1, Human Flu H3N2. The data-set used here is the same of Fig. S1.

The quantification of the degree of imbalance obtained involving both the set α, γ, η, shown in Tab. S1, and the relative behavior of $P(A)$, $P(C)$ and $C(A)$ do not allow an exhaustive discrimination among all the phylogenies. Mild differences with respect to the other phylogenies are shown for the Human Flu H3N2 tree, when considering large scale behavior ($A > 400$ and $C > 10000$).

Variants of the subtree size: ΔA

In this section we consider one possible variant of the subtree size A. In a totally balanced tree, for each internal node j, the sizes of its descending right/left subtrees (respectively A_r and A_l), are the same. In a totally imbalanced one, on the contrary, given any internal node j, one of its descending subtree, say the right one, will consist of only one leaf, thus $A_r = 1$, while $A_l = A_j - 2$. Let us consider now the variation of the subtree size ΔA, defined as:

$$\Delta A_j = A_j - A_j^*, \qquad (2)$$

where, A_j is the subtree size of the internal node j, and $A_j^* = max(A_r, A_l)$. The probability distribution of ΔA_i, also in this case, may display power law tails: $P(\Delta A) \sim \Delta A^{-\beta}$, where $\beta = 2$ for totally balanced trees, while $\beta = 0$ for totally imbalanced ones, since $\Delta A = 1$ for each internal node j.

Phylogeny	β
**totally Balanced tree**	2
**totally Imbalanced tree**	0
HIV Inter-Host	1.994
Measles Virus	1.994
Avian Flu H5N1	1.934
Human Flu H3N2	-

Table S2. β numerical values for $P(\Delta A)$ curves of Fig. S2 (Upper Panel).

In Fig. S2 (upper panel) we show the probability distribution of $P(\Delta A)$ of four RNA virus phylogenies: Human Flu H3N2, Avian Flu H5N1, Measles Virus and HIV Inter-Host. We recall that the first two phylogenies have a high expected imbalance level while this level is expected to be very low for both the Measles Virus and HIV Inter-Host phylogenies. Tab S2 shows the values β for these curves. We consider here the same data-set used in the analysis of Fig. S1.

The probability distributions $P(\Delta A)$, as well as the values of the exponent β (quantifying the asymptotical behavior of $P(\Delta A)$), is almost that of a totally balanced tree, for all the four phylogenies considered. Mild deviations are observed for the phylogeny of the Human Flu H3N2 virus, with an exceeding number of subtrees with $\Delta A > 10$, with respect to the other phylogenies.

Fig. S2 also reports (lower panel) the values ΔA^{Max} vs A for the four phylogenies under investigation. ΔA^{Max} is defined as follows. For each virus considered, we explore the relative set of 100 trees, and, among all the nodes with the same value A, we extract the one with the maximum ΔA, thus considering the maximum deviation from the imbalanced case displayed at the given size A. With this last analysis we show that phylogenies with a high expected level of imbalance (Human Flu H3N2, Avian Flu H5N1) differ from the ones expected to be balanced only on the degree of imbalance of the largest subtrees, but still, there is not a clear-cut discrimination among all the phylogenies.

Imbalance properties as a function of time: supplementary information

In order to analyze their Imbalance properties as a function of time, the phylogenetic trees of the Human Flu H3N2, Measles Virus and HIV Inter-Host have been split in sub-trees according to a temporal criterion. Each sub-tree is indeed associated with a temporal interval since all its leaves have been isolated within such an interval (See main text for further information). We give here information about the analysis of the asymptotic behavior of $\overline{M}(N)$, $\overline{d}(A)$ and $\overline{I'}(N)$ for all the temporal subtrees shown in the main text, Fig.4. We consider the extrapolation by means of the function $f(x) = alog(x)^c$, the same adopted for the analysis reported in Fig. 3 of the main text. In the Tab. S3 we report the numerical values of c.

These results attest the (asymptotic) increasing trend of the imbalance level of Human Flu H3N2 phylogeny, when considering sub-trees of longer temporal intervals. This trend is absent for the sub-trees of the HIV Intra-Host virus, while the Measles virus display a mild increasing trend, in particular when considering the extrapolation for $\overline{I'}(N)$. In this case, subtrees whose leaves have been isolated after 2004 tend to display a higher imbalance level. This behaviour can be ascribed to a non homogeneous geographical sampling of the sequences. Indeed 25/48 leaves in the year 2004 and 90/133 sequence in the year 2005 have all been isolated in the same region (China).

**Mean Topological Distance** M					
**Human Influenza A H3N2**		**HIV Inter-Host**		**Measles Virus**	
Years	c	Years	c	Years	c
1971-2001	2.024	1981-2000	1.01	1973-2000	1.277
1971-2003	2.221	1981-2002	1.02	1973-2002	1.290
1971-2005	2.326	1981-2004	1.04	1973-2004	1.387
1971-2007	2.463	1981-2006	1.02	1973-2006	1.519
1971-2011	2.741				

**Mean Depth** d					
**Human Influenza A H3N2**		**HIV Inter-Host**		**Measles Virus**	
Years	c	Years	c	Years	c
1971-2001	2.649	1981-2000	1.236	1973-2000	1.774
1971-2003	2.756	1981-2002	1.252	1973-2002	1.783
1971-2005	2.798	1981-2004	1.216	1973-2004	1.707
1971-2007	2.853	1981-2006	1.280	1973-2006	1.762
1971-2011	2.972				

**Asymmetry Metrics (I')**					
**Human Influenza A H3N2**		**HIV Inter-Host**		**Measles Virus**	
Years	c	Years	c	Years	c
1971-2001	2.185	1981-2000	0.807	1973-2000	1.566
1971-2003	2.357	1981-2002	0.823	1973-2002	1.567
1971-2005	2.456	1981-2004	0.813	1973-2004	1.828
1971-2007	2.605	1981-2006	0.821	1973-2006	1.947
1971-2011	2.697				

Table S3. asymptotical behavior of $\overline{M}(N)$, $\overline{d}(A)$ and $\overline{I'}(N)$ for the curves shown in Fig. 4, main text. In this table we report the numerical value c estimated from the extrapolation of the curves $\overline{M}(N)$, $\overline{d}(A)$ and $\overline{I'}(N)$ (Fig. 4, main text) with the functional form $f(x) = alog(x)^c$.

Metrical Properties

In this section we investigate the metrical properties of the inferred phylogenies. When inferring a phylogeny, indeed, each branch is given a length, which represents the estimated number of substitutions occurred in that branch, in the genome during the evolution corresponding to such branch. We focus here on the metrical distance of a leaf i from the *root* (MD_i) i.e., the sum of the lengths of all the branches in the path connecting i to the root. This measure is then a quantification of the substitutions accumulated in the evolutionary lineage of each leaf, with respect to the oldest common ancestor of the data-set.

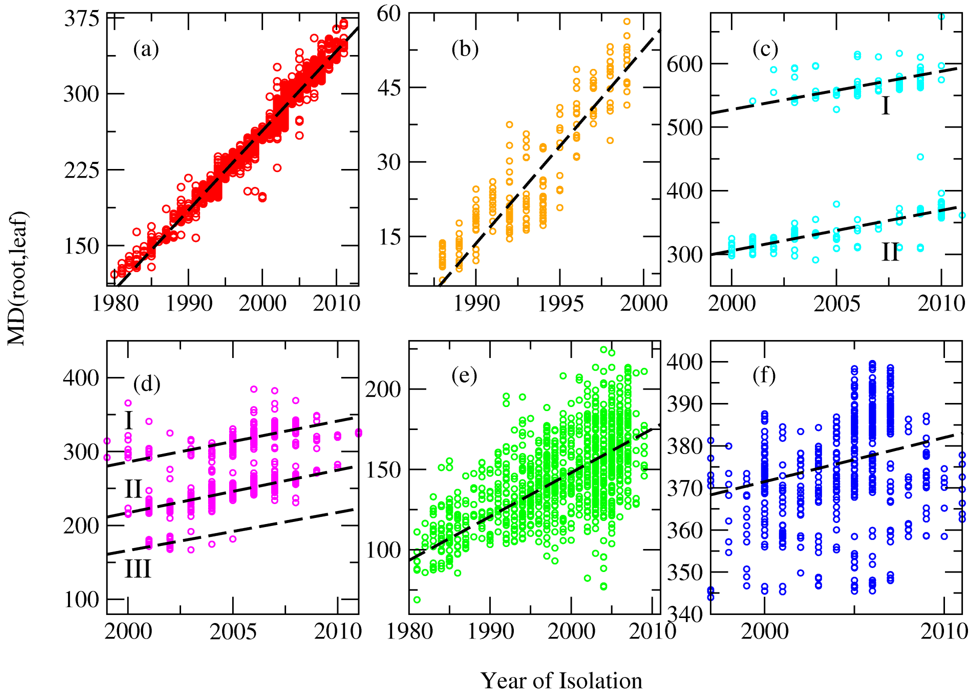

Figure S3. Metrical Properties of the phylogenies: Metrical distance (MD) of the leaves from the root of the phylogenies (a) Human Flu H3N2, (b) HIV Intra-Host, (c) Swine Flu H1N1, (d) Avian Flu H5N1, (e) HIV Inter-Host, (f) Measles Virus, as a function of the year of isolation of their leaves. Dashed lines are the linear extrapolation the MD profiles. The slope of such curves, shown in Table S4, are estimated mutation rates of the genomic regions considered to infer the phylogenies.

In Fig. S3 we show the set of MD values of the six phylogenies under analysis, as a function of the year of isolation of the leaves. The metrical properties of the inferred phylogenetic trees reflect some aspects of the evolutionary process of the viruses. The diversification process of each virus gives rise to various co-existing lineages, for each of which, the accumulation of mutations in the genome over the years turns out in the observed increasing trend for the MD. The number of observed profiles, in particular, reflects the number of co-existing lineages. The quantification of the increasing trend of each lineage can also be used to estimate the mutation rate μ of the genomic region under analysis. In Tab. S4 we report the estimated μ for each virus considered.

The analysis of the MD trends of Fig. S3 gives another evidence of the existence of one main evolutionary lineage for both the Human Flu and the HIV Intra-Host virus. The Swine Flu and the Avian Flu viruses, instead, are characterized by the co-existence of few lineages. When many lineages are co-existing, like in the Measles Virus and the HIV-Inter host virus dynamics, the MD values display a scattered behavior. In this case the values in Tab. S4 are average values for the mutational rates μ of all the coexisting lineages.

Mutation Rate

Virus	μ(substitutions/site/year)
Human Flu H3N2 (HA)	$4.63 * 10^{-3}$
HIV Intra-Host (C2V5)	$4.62 * 10^{-3}$
Swine Flu H1N1, I (HA)	$3.49 * 10^{-3}$
Swine Flu H1N1, II (HA)	$3.69 * 10^{-3}$
Avian Flu H5N1, I (HA)	$2.55 * 10^{-3}$
Avian Flu H5N1, II (HA)	$2.66 * 10^{-3}$
Avian Flu H5N1, III (HA)	$2.37 * 10^{-3}$
HIV Inter-Host (C2V5)	$2.16 * 10^{-3}$
Measles Virus (N)	$1.78 * 10^{-3}$

Table S4. Mutation Rate. Estimated mutation rate of the genomic regions used for the inference (Flu viruses: Haemagglutinin (HA), Measles Virus: N gene, HIV viruses : Env gene C2V5 of the phylogenetic trees of the viruses analyzed, derived from Fig. S3. For the Swine Flu and the Avian Flu, we report the results of the fit for each lineage (see main text for details). All these estimates are in good agreement with the mutation rates known from literature [11–13]

References

1. Mooers AO, Heard SB (1997) Inferring evolutionary process from phylogenetic tree shape. Quarterly Review of Biology 72: 31–54.

2. Matsen FA (2006) A geometric approach to tree shape statistics. Systematic Biology 55: 652–661.

3. Agapow PM, Purvis A (2002) Power of eight tree shape statistics to detect nonrandom diversification: A comparison by simulation of two models of cladogenesis. Systematic Biology 51: 866-872.

4. Fusco G, Cronk Q (1995) A new method for evaluating the shape of large phylogenies. Journal of Theoretical Biology 175: 235–243.

5. Purvis A, Agapow PM (2002) Phylogeny imbalance: Taxonomic level matters. Systematic Biology 51: 844–854.

6. Sackin MJ (1972) Good and bad phenograms. Systematic Biology 21: 225–226.

7. Shao K, Sokal R (1990) Tree balance. Syst Zool 39: 266–276.

8. Herrada EA, Tessone CJ, Eguíluz VM, Hernández-García E, Duarte CM (2008) Universal scaling in the branching of the tree of life. PLoS ONE 3: e2757.

9. Stich M, Manrubia SC (2009) Topological properties of phylogenetic trees in evolutionary models. European Physical Journal B 70: 583–592.

10. Harris TE (1963) The theory of branching processes. Courier Dover Publications.

11. Oldstone MB, Dales S, Tishon A, Lewicki H, Martin L (2005) A role for dual viral hits in causation of subacute sclerosing panencephalitis. The Journal of Experimental Medicine 202: 1185–1190.

12. Lemey P, Rambaut A, Pybus G (2006) Hiv evolutionary dynamics within and among hosts. AIDS Reviews 3: 125–140.

13. Fitch WM, Bush RM, Bender CA, Cox NJ (1997) Long term trends in the evolution of h(3) ha1 human influenza type a. Proceedings of The National Academy of Sciences 94: 7712–7718.
